# Prion disease pathogenesis in the absence of the commensal microbiota

**DOI:** 10.1099/jgv.0.000860

**Published:** 2017-07-15

**Authors:** Barry M. Bradford, Laura Tetlow, Neil A. Mabbott

**Affiliations:** The Roslin Institute and Royal (Dick) School of Veterinary Sciences, University of Edinburgh, Easter Bush EH25 9RG, UK

**Keywords:** transmissible spongiform encephalopathies, microbiota, brain, central nervous system, CNS, microglia

## Abstract

Prion diseases are a unique group of transmissible, typically sub-acute, neurodegenerative disorders. During central nervous system (CNS) prion disease, the microglia become activated and are thought to provide a protective response by scavenging and clearing prions. The mammalian intestine is host to a large burden of commensal micro-organisms, especially bacteria, termed the microbiota. The commensal microbiota has beneficial effects on host health, including through the metabolism of essential nutrients, regulation of host development and protection against pathogens. The commensal gut microbiota also constitutively regulates the functional maturation of microglia in the CNS, and microglial function is impaired when it is absent in germ-free mice. In the current study, we determined whether the absence of the commensal gut microbiota might also affect prion disease pathogenesis. Our data clearly show that the absence of the commensal microbiota in germ-free mice did not affect prion disease duration or susceptibility after exposure to prions by intraperitoneal or intracerebral injection. Furthermore, the magnitude and distribution of the characteristic neuropathological hallmarks of terminal prion disease in the CNS, including the development of spongiform pathology, accumulation of prion disease-specific protein (PrP), astrogliosis and microglial activation, were similar in conventionally housed and germ-free mice. Thus, although the commensal gut microbiota constitutively promotes the maintenance of the microglia in the CNS under steady-state conditions in naïve mice, our data suggest that dramatic changes to the abundance or complexity of the commensal gut microbiota are unlikely to influence CNS prion disease pathogenesis.

## Abbreviations

BSE, bovine spongiform encephalopathy; CNS, central nervous system; FDC, follicular dendritic cell; GALT, gut-associated lymphoid tissues; GFAP, glial fibrillary acidic protein; IC, intracerebral; IHC, immunohistochemistry; ILF, isolated lymphoid follicle; IP, intraperitoneal; PrP, prion protein; SCFA, short chain fatty acid; SPF, specific pathogen free; vCJD, variant Creutzfeldt-Jakob disease.

## Introduction

Prion diseases, or transmissible spongiform encephalopathies, are typically sub-acute neurodegenerative diseases that affect humans and a number of captive and free-ranging animal species. During prion disease progression, affected tissues accumulate abundant aggregations of PrP^Sc^, abnormally folded isoforms of the host-encoded cellular prion protein, PrP^C^ [1]. Cumulative scientific evidence suggests that PrP^Sc^ is the major, if not sole, component of the infectious prion agent [2, 3]. Once the prions establish infection in the central nervous system (CNS) they often cause extensive neuropathology that is typically characterised by the activation of microglia and astrocytes, accumulations of PrP^Sc^, and neurodegeneration [4, 5].

Microglia are the tissue macrophages of the CNS and play important roles in maintaining neuronal homeostasis, synaptic remodelling, clearing dead and dying cells, and providing a first line of defence against pathogens [6–9]. A change in microglial status, from resting to activated, is amongst the first pathological features observed during CNS prion disease, occurring before the onset of neuropathology [4, 10]. Within the prion disease-affected brain, an anti-inflammatory cytokine milieu appears to be induced in order to stimulate the microglia to protect the host by scavenging and clearing prions and prion-affected cells [11–15]. However, systemic administration of inflammatory stimuli such as bacterial lipopolysaccharide (LPS) during CNS prion disease can switch the microglial phenotype to one with a more pro-inflammatory bias, leading to exacerbated neuropathology [16, 17]. Consistent with this hypothesis, prion disease is delayed in mice deficient in CD14 (a component of the LPS receptor) and is accompanied by enhanced expression of anti-inflammatory cytokines such as IL-10 [18].

The mammalian gastrointestinal tract is home to a vast community of commensal micro-organisms, termed the microbiota [19]. For example, the large intestine of the adult human may contain approximately 100 trillion micro-organisms, representing over 1000 different bacterial species. The commensal gut microbiota provides a diverse range of beneficial effects to host health including the metabolism of essential nutrients [20], influencing the development and regulation of the immune system [21, 22], and providing protection against pathogens by outcompeting them for nutrients or habitats [23].

A bidirectional neurohumoral communication system, termed the gut–brain axis, can integrate neural, hormonal and immunological signalling between these two tissues. This enables the brain to influence a variety of physiological activities in the intestine including motility and secretion, and the actions of the mucosal immune system [24]. Gut- and microbiota-derived products can also influence the brain, for example, through the release of cytokines or hormones from enteroendocrine cells, or via stimulation of afferent neural pathways of the vagus nerve and spinal cord. These, in turn, can influence the composition of the gut microbiota, either directly, or due to physiological effects on the intestine (for reviews see [24–27]). Exciting data have revealed that the commensal gut microbiota constitutively maintains the homeostasis of the microglia in the brains of naïve mice under steady-state conditions [28]. Furthermore, the functional maturation of the microglia was compromised in germ-free mice and coincided with their significantly reduced responses to LPS-stimulation or virus infection in the CNS [28]. The nature of the microglial activation status during CNS prion disease is considered to influence the severity of neurodegeneration [5], but whether the commensal gut microbiota also influence prion disease pathogenesis is uncertain [29]. Modifications to the gut microbiota can contribute to the pathogenesis of anxiety and depression, and certain neurodegenerative disorders such as Alzheimer’s disease and Parkinson’s disease [30]. Therefore, in the current study, prion disease pathogenesis and susceptibility were compared in conventional and germ-free mice to determine whether significant reductions to the abundance of the commensal gut microbiota might also affect prion disease pathogenesis and susceptibility.

## Results

Groups of conventionally housed mice (specific pathogen free control) and germ-free mice were injected with prions by either intracerebral (IC; *n*=10/group) or intraperitoneal (IP; *n*=9/group) routes and survival times and disease incidence compared. As anticipated, all the conventionally housed mice succumbed to clinical prion disease with a mean survival time of 174±2 days after IC exposure (median 175 days), and 248±3 days after IP exposure (median 247 days; Table 1). Our data show that the microbiological status of the mice did not significantly affect survival times or disease susceptibility as all the germ-free mice succumbed to clinical disease with similar survival times when injected with prions by either of these exposure routes (IC, mean 174±0 days, median 175 days, *P*=0.702; IP, mean 245±3 days, median 245 days, *P*=0.576). These data clearly demonstrate that the absence of the commensal microbiota in germ-free mice does not influence disease susceptibility or duration when infected with prions directly into the CNS (IC) or after peripheral exposure via the peritoneal cavity (IP).

**Table 1.** Prion disease susceptibility in conventional and germ-free mice

Group	Route of prion exposure*	Individual survival times (days)	Mean survival times (days ±se)	Clinical disease†	Vacuolar pathology in brain	*P-*value‡
Conventional	IC	163, 168, 2×173, 5×175, 185	174±2	10/10	10/10	
Germ-free	IC	3×173, 7×175	174±0	10/10	10/10	0.702
Conventional	IP	2×238, 2×245, 249, 252, 255, 257, 261	248±3	9/9	9/9	
Germ-free	IP	235, 2×236, 3×245, 248, 258, 261	245±3	9/9	9/9	0.576

*Mice were injected IC or IP with 20 µl of a 1.0 % dose of mouse-passaged 22C scrapie prions.

†Incidence = no. animals affected/no. animals tested.

‡Conventional versus germ-free, Student’s *t*-test.

**Fig. 1.**
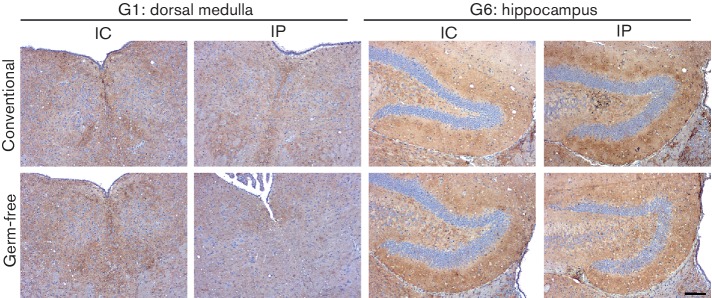
Influence of the commensal gut microbiota on the distribution of prion disease-specific PrP within the brains of mice with terminal prion disease. Conventionally housed or germ-free mice were injected with 22C prions by the IC or IP routes, and brains were collected at the terminal stage of disease. Sections were immunostained to detect PrP (brown) and counterstained with haematoxylin (blue) to detect cell nuclei. IHC analysis suggested that the absence of the commensal gut microbiota in germ-free mice did not influence the distribution of disease-specific PrP within the brain. Representative images are presented from six mice per group. Scale bar, 100 µm.

**Fig. 2.**
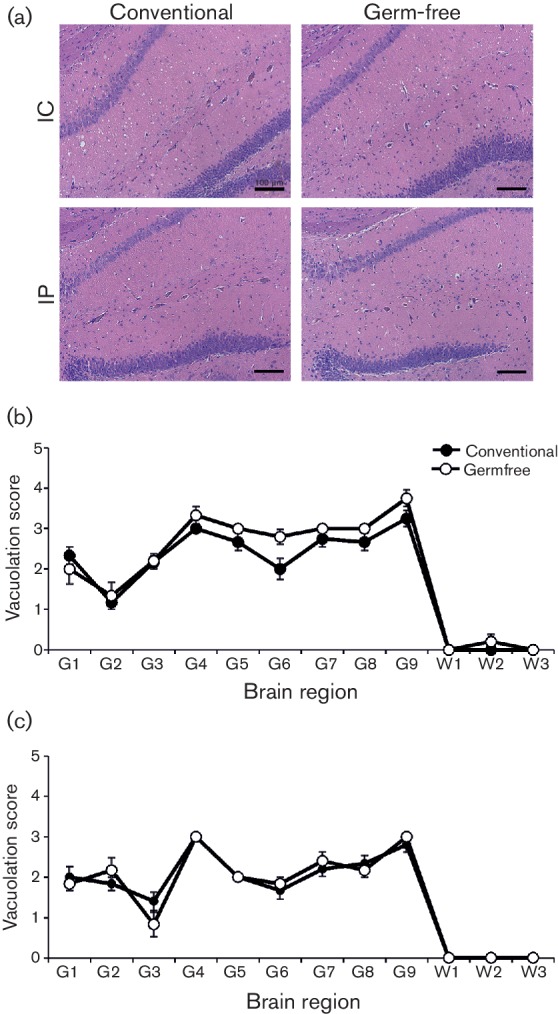
The commensal gut microbiota does not influence the magnitude or distribution of the spongiform pathology within the brain at the terminal stage of prion disease. Conventionally housed or germ-free mice were injected with prions by the IC (*n*=10/group) or IP (*n*=9/group) routes, and brains collected at the terminal stage of disease. (a) High levels of spongiform pathology were detected in the brains of all the conventionally housed and germ-free mice with terminal prion disease. Sections were stained with haematoxylin and eosin. Scale bar, 100 µm. Mice were infected with prions by the IC (b) or IP (c) routes, and at the terminal stage of the disease the severity and distribution of the spongiform pathology (vacuolation) within each brain was scored on a scale of one to five in nine grey matter areas: G1, dorsal medulla; G2, cerebellar cortex; G3, superior colliculus; G4, hypothalamus; G5, thalamus; G6, hippocampus; G7, septum; G8, retrosplenial and adjacent motor cortex; G9, cingulate and adjacent motor cortex. Each point represents the mean vacuolation score±se. IC (*n*=10/group) or IP (*n*=9/group).

During prion diseases, aggregations of prion disease-specific PrP (PrP^d^) accumulate in affected tissues. Immunohistochemistry (IHC) analysis suggested that the abundances of prion disease-specific PrP^d^ within the brains of clinically affected conventionally housed or germ-free mice were similar (Fig. 1). Prion infection within the CNS also leads to the development of significant spongiform pathology (vacuolation) in the neuropil (Fig. 2a). We therefore compared the magnitude of the spongiform pathology within nine distinct grey matter regions in the brains of all the clinically affected mice from each group. Irrespective of the microbiological status of the mice, the average severity and distribution of the spongiform pathology within the brains of the clinically affected mice after IC or IP injection with prions were similar (Fig. 2b, c, respectively). These data clearly show that the absence of the commensal microbiota in germ-free mice did not affect the accumulation of PrP^d^ in the brain or the development of spongiform pathology at the clinical stage of disease.

**Fig. 3.**
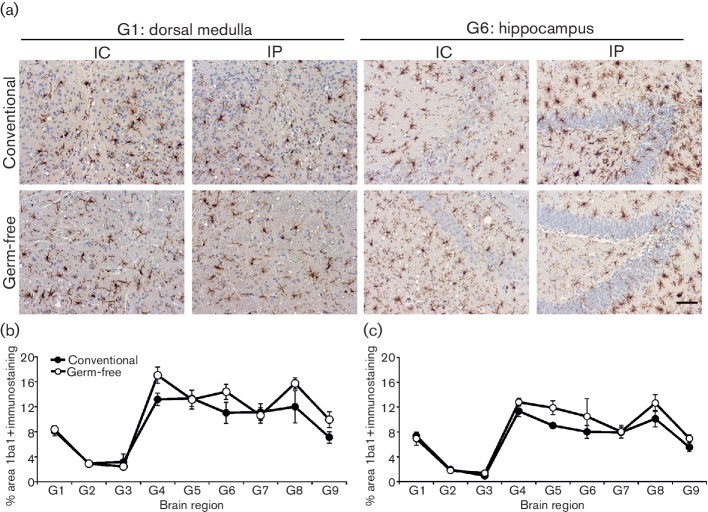
Influence of the commensal gut microbiota on the distribution of microglia within the brains of mice with terminal prion disease. Conventionally housed or germ-free mice were injected with prions by the IC or IP routes and brains collected at the terminal stage of disease. (a) Sections were immunostained to detect microglia (Iba1^+^ cells, brown) and counterstained with haematoxylin (blue) to detect cell nuclei. Scale bar, 100 µm. Morphometric analysis revealed that the area of the Iba1^+^ immunostaining in nine distinct grey matter regions (regions as in the legend to Fig. 2) was similar in the brains of clinically affected conventionally housed and germ-free mice infected with prions by the IC (b) or IP (c) routes. *n*=6 mice/group.

Microglial activation is another prominent histopathological feature within the prion disease-affected brain [4, 5, 10]. Since the commensal microbiota can constitutively regulate the functional maturation of the microglia [28], we next compared the status of the microglia in the brains of conventionally housed and germ-free mice with clinical prion disease. Our IHC analysis suggested a similar abundance of Iba1^+^ microglia in the brains of the clinically affected conventionally housed and germ-free mice after IC or IP injection with prions (Fig. 3a). Morphometric analysis of nine distinct grey matter regions also indicated equivalent areas of Iba1^+^ immunostaining in the brains of mice from each group (Fig. 3b, IC-injected; Fig. 3c, IP-injected).

**Fig. 4.**
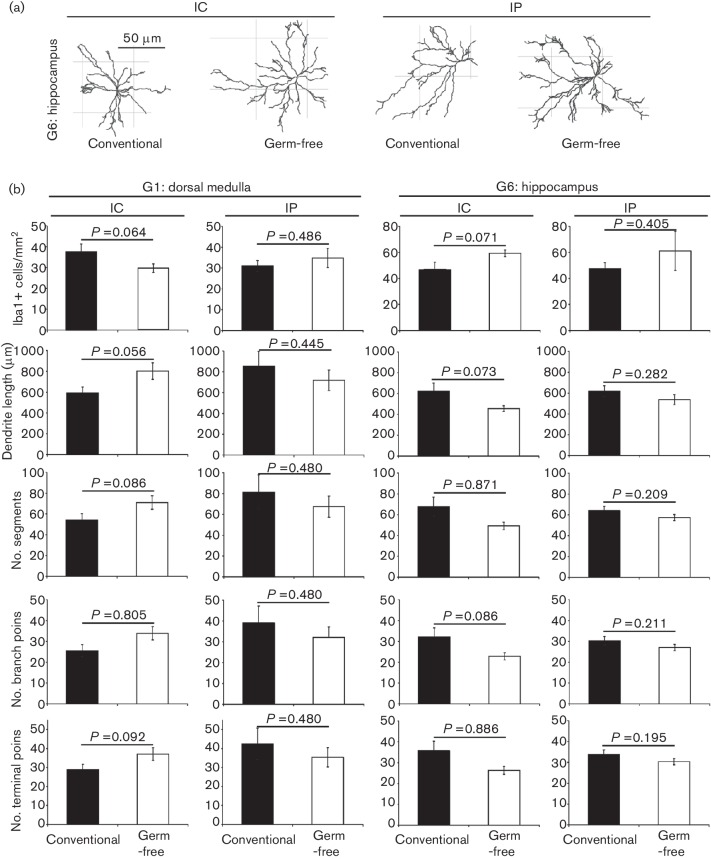
Semi-automatic quantitative morphometric analysis of the density and morphology of microglia in the brains of conventionally housed or germ-free mice with terminal prion disease. (a) Representative Imaris-based reconstructions of Iba1^+^ microglia in the hippocampus region of mice from each group. (b) Imaris-based quantitative cell morphometry of Iba1^+^ microglia in the dorsal medulla (left-hand panels) and hippocampus (right-hand panels) regions. Six individual mice from each mouse group were sampled, and from each of their brains, data were collected from at least 25 individual Iba1^+^ microglia within each brain region. Solid bars, conventionally housed mice; open bars, germ-free mice. No significant differences between groups were observed in the number of microglia within regions, or in the length of dendrites, number of segments, branching points or terminal points on individual microglia.

Independent histopathological analyses have demonstrated increased microglial size and densities in the brains of naïve germ-free mice [28]. A similar semi-automatic quantitative morphometric analysis approach was used here to compare the density and morphology of the Iba1^+^ microglia in the brains of conventionally housed and germ-free mice with clinical prion disease. From each group, six individual mice were sampled, and from each of their brains, data were collected from at least 25 individual Iba1^+^ microglia within the dorsal medulla (G1) and hippocampus (G6) regions. Our data clearly show that the density and morphology of the microglia within each region were similar in brains from clinically affected conventionally housed or germ-free mice (Fig. 4). No significant differences between groups were observed in the number of microglia within regions, or in the length of dendrites, number of segments, branching points or terminal points on individual microglia (Fig. 4b). Thus although the commensal gut microbiota may control the homeostasis of the microglia under steady-state conditions in naïve mice [28], our data show that its absence does not significantly affect the density or morphology of microglia during CNS prion disease.

**Fig. 5.**
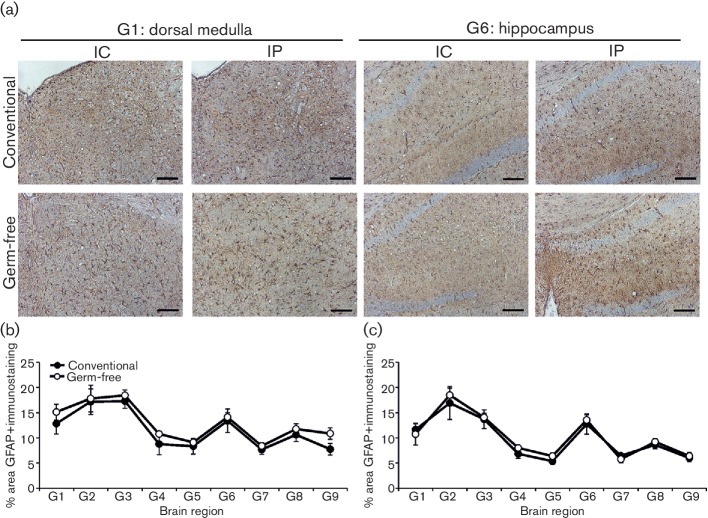
Influence of the commensal gut microbiota on the distribution of reactive astrocytes within the brains of mice with terminal prion disease. Conventionally housed or germ-free mice were injected with prions by the IC or IP routes, and brains collected at the terminal stage of disease. (a) Sections were immunostained to detect astrocytes (GFAP^+^ cells, brown) and counterstained with haematoxylin (blue) to detect cell nuclei. Scale bar, 100 µm. Morphometric analysis revealed that the area of the GFAP^+^ immunostaining in nine distinct grey matter regions (regions as in the legend to Fig. 2) was similar in the brains of clinically affected conventionally housed and germ-free mice infected with prions by the IC (b) or IP (c) routes. *n*=6 mice/group.

CNS prion disease is also accompanied by the induction of reactive astrocytes expressing high levels of glial fibrillary acidic protein (GFAP). Our IHC analysis showed that the abundance of GFAP^+^ immunostaining across nine distinct grey matter regions was similar in the brains of conventionally housed or germ-free mice at the terminal stage of prion disease (Fig. 5). These data are consistent with the independent demonstration that the number of GFAP^+^ astrocytes in the brain was not affected by the absence of the commensal microbiota in naïve germ-free mice [28].

## Discussion

The commensal gut microbiota can constitutively regulate the functional maturation of the microglia in the CNS and influence their responses to LPS-stimulation or CNS virus infection [28]. Disturbances to the gut microbiota can contribute to the pathogenesis of several human neurodegenerative diseases [30]. Microglial activation is a prominent feature of CNS prion disease [4, 10], but whether the commensal gut microbiota influence the progression of CNS disease is uncertain [29, 31, 32]. Data in the current study clearly demonstrate that prion disease pathogenesis and susceptibility after exposure by either the IP or IC routes is not influenced by the absence of the commensal gut microbiota in germ-free mice. We also show that the microbiological status of the host did not influence the magnitude or distribution of the characteristic neuropathological hallmarks of terminal prion disease in the CNS, including the development of spongiform pathology, accumulation of disease-specific PrP, astrogliosis and microgliosis. Thus, these data suggest that dramatic changes to the abundance or complexity of the commensal gut microbiota are unlikely to influence prion disease duration or susceptibility, or the development of neuropathology in the CNS. Of course, our data cannot exclude the possibility that an effect of the microbiota on CNS disease pathogenesis may have been observed after exposure of the germ-free mice to a much lower dose of prions than used in the current study.

Data presented in the current study appear to contradict data reported in two independent studies reported more than 20 years ago [31, 32]. However, fundamental differences in experimental design and purpose make direct comparisons between these studies difficult. In an attempt to define the nature of the scrapie prion agent, Lev and colleagues infected conventionally housed and germ-free CD1 mice with lyophylized and irradiated preparations of Chandler mouse-adapted scrapie isolate by the IC route. Survival times were reported to be extended in the germ-free mice, but from the data presented it is uncertain whether this effect was significant [31]. In a later study, Wade and colleagues compared prion disease survival times after IC or IP injection with ME7 scrapie prions in germ-free mice with those colonized with a highly restricted Gram-positive bacterial microflora [32]. Consistent with data presented in the current study, survival times after IC prion exposure were not influenced by the microbiological status of the mice, but a prolongation was observed in the germ-free mice after IP exposure [32]. Precise explanations for the inconsistencies between data in this study and the current study are uncertain, but a highly restricted Gram-positive bacterial microflora similar to that used as a control in the Wade study can itself influence microglial status [28]. After IP exposure to prions, early accumulation and replication of the prions on follicular dendritic cells (FDCs) within the B cell follicles of the spleen is essential for them to subsequently infect the CNS, termed neuroinvasion [33–35]. Certain bacterial constituents of the commensal gut microbiota can have a profound influence on the function and development of the host immune system [22, 36, 37]. Therefore, the possibility cannot be excluded that a highly restricted Gram-positive bacterial flora may have enhanced prion neuroinvasion from the spleen after IP exposure, shortening survival times when compared to germ-free mice.

The study by Erny and colleagues [28] showed that the pro-inflammatory responses of microglia after LPS stimulation were diminished in the brains of germ-free mice. The microglial phenotype during CNS prion disease, in contrast, is considered to be predominantly anti-inflammatory to enable the microglia to protect the host by scavenging and clearing prions [11–15]. In the current study, our data show that microglial abundance and complexity in the brains of mice with clinical prion disease were unaltered in the absence of the microbiota. Effects of the microbiota on the microglial response to anti-inflammatory stimuli were not determined in the above study [28]. However, it is plausible that the influence of the microbiota on microglia may be dependent on their activation status and phenotype.

Many of the actions of the commensal microbiota on the host immune system have been shown to be mediated through the ability of some bacterial species to break down indigestible dietary fibre into short chain fatty acids (SCFAs) [22]. Furthermore, microglial development can be restored in germ-free mice by the administration of SCFAs [28]. The human ‘Western diet’ is characteristically high in fat and simple carbohydrates, and can modify the constituents of the commensal gut microbiota by decreasing the abundance of Bacteroidetes, which are important providers of SCFA [38, 39]. Despite its potential to induce significant changes in the commensal gut microbiota, CNS prion pathogenesis is not significantly affected in mice raised on a high-fat diet [40].

The replication of certain prion strains on FDCs in the gut-associated lymphoid tissue (GALT) in the small intestine (such as the Peyer’s patches and isolated lymphoid follicles, ILFs) is essential for efficient neuroinvasion after oral exposure [41–45]. Although data in the current study suggest that dramatic changes to the abundance or complexity of the commensal gut microbiota do not influence CNS prion disease pathogenesis or susceptibility after IC or IP exposure, potential effects on susceptibility to orally acquired prion infections cannot be excluded. Cross-talk between the host immune system and the commensal microbiota can significantly influence the development and function of the GALT [36, 37, 46]. Mature ILFs, comprising individual B cell follicles with a network of FDCs, can act as important sites of prion accumulation and neuroinvasion in the small intestine [43, 44]. The density of ILFs is reduced in the small intestines of germ-free mice [46], implying that microbiota-mediated alterations to GALT abundance or status in the small intestine could significantly affect oral prion disease susceptibility by influencing the uptake, replication and neuroinvasion of prions from the gut lumen. Further studies are clearly necessary to study the effects of the commensal gut microbiota on oral prion disease susceptibility. However, the use of germ-free mice is not appropriate here as these mice have altered gastrointestinal motility and transit time [47] and substantially enlarged caeca [48], which could have profound effects on the uptake of prions from the gut lumen after oral exposure.

Although changes to the abundance or complexity of the commensal gut microbiota are unlikely to influence prion disease duration or susceptibility, concurrent gastrointestinal infection with pathogenic bacteria or systemic exposure to their pro-inflammatory components can exacerbate prion disease pathogenesis. For example, systemic treatment with bacterial LPS can induce an aggressive pro-inflammatory phenotype in the microglia in the prion disease affected brain, leading to increased neuropathology and accelerated disease progression [49, 50]. Pathology to the gut epithelium at the time of oral prion exposure, such as that caused by oral infection with the enteroinvasive bacterium *Salmonella* *typhimurium*, can also increase prion disease susceptibility [51], most likely through the enhanced uptake of prions from the gut lumen [52].

Despite the apparent widespread exposure of the UK human population to bovine spongiform encephalopathy (BSE) prions through the food chain [53, 54], it is fortunate that relatively low numbers of clinical cases of variant Creutzfeldt–Jakob disease (vCJD) in humans have been reported [55]. However, data from the retrospective analyses of PrP^Sc^ accumulation in archived human GALT specimens suggest the prevalence of vCJD infection may be higher than the clinical cases suggest [56]. This implies the potential existence of a subclinical carrier state. A thorough understanding of the factors which influence the progression of the preclinical phase will help to identify those that enhance the risk of developing clinical prion disease. For example, the presence of prion-specific PrP^Sc^ within specific regions of the brains of prion-affected hosts is insufficient on its own to trigger neurodegeneration [5]. Neurodegeneration was observed to occur only when PrP^Sc^ accumulation was associated with an upregulated innate immune response by the microglia within the same brain regions. This indicates that factors manipulating the activation status of the microglial may have a significant impact on the magnitude of the neurodegeneration exhibited during CNS prion disease. For example, factors that enhance clearance of prions by microglia may reduce disease pathogenesis. Conversely, factors that enhance the destruction of neurones by microglia may enhance the progression of CNS prion disease [49, 50]. However, although the commensal gut microbiota can control the functional maturation of microglia, data presented in the current study suggest that dramatic changes to the commensal microbiota are unlikely to influence the development of CNS prion disease.

## Methods

### Mice

C3H/LacDk mice (6–10 weeks old) were used throughout this study. Where indicated, some mice were conventionally housed and maintained under standard specific pathogen free (SPF) conditions. Others were bred and maintained under strict germ-free conditions in sterile isolators. SPF mice and germ free mice were each housed in groups of four to six in conventional cages with sterilized Aspen Chip bedding, under a 12 h light/12 h dark cycle, and provided with unrestricted access to sterile water and food (RM1, Special Diet Services, Essex, UK). The germ-free status of the colony was continually maintained over many years and confirmed by regular microbial testing of sentinel mice from the same isolators. Indeed, in separate studies our analyses confirmed that germ-free mice from our colony had the disturbed development of gut-associated lymphoid tissues in their intestines and substantially enlarged caeca [46] reported in independent studies of germ-free mice elsewhere [48, 57, 58]. All studies and regulatory licences were approved by the Neuropathogenesis Unit’s ethics committee and carried out under the authority of a UK Home Office Project Licence.

### Prion exposure and disease monitoring

Where indicated, mice were injected by either the intracerebral (IC) or intraperitoneal (IP) routes with 20 µl of a 1.0 % (w/v) dilution of scrapie brain homogenate prepared from mice terminally affected with 22C scrapie prions (containing approximately 4.5 log IC infectious dose_50_ units). The brains used to prepare the homogenate used in this study were washed in absolute alcohol and γ-irradiated before use to eliminate potential contamination from commensal micro-organisms. Following prion exposure, mice were coded and assessed weekly for signs of clinical disease and culled at a standard clinical endpoint. The clinical endpoint of disease was determined by rating the severity of the clinical signs of prion disease exhibited by the mice. Mice were clinically scored as ‘unaffected’, ‘possibly affected’ and ‘definitely affected’ according to the standard clinical characteristics typically present in mice with terminal scrapie prion disease. Clinical signs following infection of mice with scrapie prions may include: weight loss, ruffled or starry/spiky fur, hunched posture, jumpy behaviour (at early onset) progressing to limited movement, upright tail, wet genitals, decreased awareness, discharge from eyes/blinking eyes and ataxia of hind legs. The clinical endpoint of disease was defined in one of the following ways: (i) the day on which a mouse received a second consecutive ‘definite’ rating; (ii) the day on which a mouse received a third ‘definite’ rating within four consecutive weeks; (iii) the day on which a mouse was culled *in extremis*. Survival times were recorded for mice that did not develop clinical signs of disease during the observation period. Prion diagnosis was confirmed by histopathological assessment of vacuolation in the brain. For the construction of lesion profiles, vacuolar changes were scored in nine distinct grey-matter regions of the brain as described previously [59].

### Immunohistochemisty (IHC)

For the detection of prion disease-specific PrP (PrP^d^), brains were fixed in paraformaldehyde fixative and embedded in paraffin wax. Sections (thickness, 6 µm) were deparaffinized and pre-treated to enhance the detection of PrP^d^ by hydrated autoclaving (15 min, 121 °C, hydration) and subsequent immersion in formic acid (98 %) for 5 min. Sections were then immunostained with PrP-specific mAb BH1 [60]. For the detection of astrocytes, brain sections were immunostained with anti-glial fibrillary acidic protein (GFAP; DAKO, Ely, UK). For the detection of microglia, deparaffinized brain sections were first pre-treated with citrate buffer and subsequently immunostained with anti-ionized calcium-binding adaptor molecule 1 (Iba1; Wako Chemicals GmbH, Neuss, Germany). Following the addition of primary antibodies, biotin-conjugated species-specific secondary antibodies (Stratech, Soham, UK) were applied and immunolabelling was revealed using horseradish peroxidase conjugated to the avidin–biotin complex (ABC kit, Vector Laboratories, Peterborough, UK) and visualized with 3,3′-diaminobenzidine (Sigma). Sections were counterstained with haematoxylin to distinguish cell nuclei.

### Image analysis

For morphometric analysis, images were analysed using ImageJ software (http://rsb.info.nih.gov/ij/) as described on coded sections [61]. Background intensity thresholds were first applied using an ImageJ macro which measures pixel intensity across all immunostained and non-stained areas of the images. The obtained pixel intensity threshold value was then applied in all subsequent analyses. Next, the number of pixels of each colour were automatically counted. For these analyses, data are presented as the proportion of positively stained pixels for a given IHC marker per total number of pixels (all colours) in the specific area of interest. In each instance tissues from six mice per group were analysed. Details of all the sample sizes for each parameter analysed are provided in the figure legends.

The density and morphology of Iba1^+^ microglia was analysed using Imaris 8.2.1 software (Bitplane, Belfast, UK) according to a previously established protocol [28]. Six individual mice were sampled from each group and from each of these mice data were collected from at least 25 individual Iba1^+^ microglia within the dorsal medulla (G1) and hippocampus (G6) regions of their brains. Microglial morphology was compared by measuring dendrite length, numbers of segments, branching and terminal points. Thus, from each mouse group, data were collected from at least 150 individual Iba1^+^ microglia.

### Statistical analyses

Unless indicated otherwise, data are presented as mean±se and significant differences between groups were sought by using Student’s *t*-test. Values of *P*<0.05 were accepted as significant.

## References

[1] Prusiner SB, Bolton DC, Groth DF, Bowman KA, Cochran SP (1982). Further purification and characterization of scrapie prions. Biochemistry.

[2] Legname G, Baskakov IV, Nguyen HO, Riesner D, Cohen FE (2004). Synthetic mammalian prions. Science.

[3] Wang F, Wang X, Yuan CG, Ma J (2010). Generating a prion with bacterially expressed recombinant prion protein. Science.

[4] Vincenti JE, Murphy L, Grabert K, Mccoll BW, Cancellotti E (2015). Defining the microglia response during the time course of chronic neurodegeneration. J Virol.

[5] Alibhai J, Blanco RA, Barria MA, Piccardo P, Caughey B (2016). Distribution of misfolded prion protein seeding activity alone does not predict regions of neurodegeneration. PLoS Biol.

[6] Kranich J, Krautler NJ, Falsig J, Ballmer B, Li S (2010). Engulfment of cerebral apoptotic bodies controls the course of prion disease in a mouse strain–dependent manner. J Exp Med.

[7] Zhan Y, Paolicelli RC, Sforazzini F, Weinhard L, Bolasco G (2014). Deficient neuron-microglia signaling results in impaired functional brain connectivity and social behavior. Nat Neurosci.

[8] Prinz M, Priller J (2014). Microglia and brain macrophages in the molecular age: from origin to neuropsychiatric disease. Nat Rev Neurosci.

[9] de Lucia C, Rinchon A, Olmos-Alonso A, Riecken K, Fehse B (2016). Microglia regulate hippocampal neurogenesis during chronic neurodegeneration. Brain Behav Immun.

[10] Williams A, Lucassen PJ, Ritchie D, Bruce M (1997). PrP deposition, microglial activation, and neuronal apoptosis in murine scrapie. Exp Neurol.

[11] Boche D, Cunningham C, Gauldie J, Perry VH (2003). Transforming growth factor-β1-mediated neuroprotection against excitotoxic injury *in vivo*. J Cereb Blood Flow Metab.

[12] Minghetti L, Greco A, Cardone F, Puopolo M, Ladogana A (2000). Increased brain synthesis of prostaglandin E2 and F2-isoprostane in human and experimental transmissible spongiform encephalopathies. J Neuropathol Exp Neurol.

[13] Walsh DT, Perry VH, Minghetti L (2000). Cyclooxygenase-2 is highly expressed in microglial-like cells in a murine model of prion disease. Glia.

[14] Cunningham C, Deacon R, Wells H, Boche D, Waters S (2003). Synaptic changes characterize early behavioural signs in the ME7 model of murine prion disease. Eur J Neurosci.

[15] Zhu C, Herrmann US, Falsig J, Abakumova I, Nuvolone M (2016). A neuroprotective role for microglia in prion diseases. J Exp Med.

[16] Combrinck MI, Perry VH, Cunningham C (2002). Peripheral infection evokes exaggerated sickness behaviour in pre-clinical murine prion disease. Neuroscience.

[17] Cunningham C, Wilcockson DC, Campion S, Lunnon K, Perry VH (2005). Central and systemic endotoxin challenges exacerbate the local inflammatory response and increase neuronal death during chronic neurodegeneration. J Neurosci.

[18] Sakai K, Hasebe R, Takahashi Y, Song CH, Suzuki A (2013). Absence of CD14 delays progression of prion diseases accompanied by increased microglial activation. J Virol.

[19] Sommer F, Bäckhed F (2013). The gut microbiota**—**masters of host development and physiology. Nat Rev Microbiol.

[20] Russell WR, Hoyles L, Flint HJ, Dumas ME (2013). Colonic bacterial metabolites and human health. Curr Opin Microbiol.

[21] Hooper LV, Littman DR, Macpherson AJ (2012). Interactions between the microbiota and the immune system. Science.

[22] Furusawa Y, Obata Y, Fukuda S, Endo TA, Nakato G (2013). Commensal microbe-derived butyrate induces the differentiation of colonic regulatory T cells. Nature.

[23] Kamada N, Kim YG, Sham HP, Vallance BA, Puente JL (2012). Regulated virulence controls the ability of a pathogen to compete with the gut microbiota. Science.

[24] Mayer EA (2011). Gut feelings: the emerging biology of gut-brain communication. Nat Rev Neurosci.

[25] Collins SM, Surette M, Bercik P (2012). The interplay between the intestinal microbiota and the brain. Nat Rev Microbiol.

[26] Bauer PV, Hamr SC, Duca FA (2016). Regulation of energy balance by a gut–brain axis and involvement of the gut microbiota. Cell Mol Life Sci.

[27] Erny D, Hrabě de Angelis AL, Prinz M (2017). Communicating systems in the body: how microbiota and microglia cooperate. Immunology.

[28] Erny D, Hrabě de Angelis AL, Jaitin D, Wieghofer P, Staszewski O (2015). Host microbiota constantly control maturation and function of microglia in the CNS. Nat Neurosci.

[29] Donaldson DS, Mabbott NA (2016). The influence of the commensal and pathogenic gut microbiota on prion disease pathogenesis. J Gen Virol.

[30] Fung TC, Olson CA, Hsiao EY (2017). Interactions between the microbiota, immune and nervous systems in health and disease. Nat Neurosci.

[31] Lev M, Raine CS, Levenson SM (1971). Enhanced survival of germfree mice after infection with irradiated scrapie brain. Experientia.

[32] Wade WF, Dees C, German TL, Marsh RF (1986). Effect of bacterial Flora and mouse genotype (euthymic or athymic) on scrapie pathogenesis. J Leukoc Biol.

[33] Mabbott NA, Mackay F, Minns F, Bruce ME (2000). Temporary inactivation of follicular dendritic cells delays neuroinvasion of scrapie. Nat Med.

[34] Montrasio F, Frigg R, Glatzel M, Klein MA, Mackay F (2000). Impaired prion replication in spleens of mice lacking functional follicular dendritic cells. Science.

[35] Mcculloch L, Brown KL, Bradford BM, Hopkins J, Bailey M (2011). Follicular dendritic cell-specific prion protein (PrP^C^) expression alone is sufficient to sustain prion infection in the spleen. PLoS Pathog.

[36] Obata T, Goto Y, Kunisawa J, Sato S, Sakamoto M (2010). Indigenous opportunistic bacteria inhabit mammalian gut-associated lymphoid tissues and share a mucosal antibody-mediated symbiosis. Proc Natl Acad Sci USA.

[37] Lécuyer E, Rakotobe S, Lengliné-Garnier H, Lebreton C, Picard M (2014). Segmented filamentous bacterium uses secondary and tertiary lymphoid tissues to induce gut IgA and specific T helper 17 cell responses. Immunity.

[38] Turnbaugh PJ, Ridaura VK, Faith JJ, Rey FE, Knight R (2009). The effect of diet on the human gut microbiome: a metagenomic analysis in humanized gnotobiotic mice. Sci Transl Med.

[39] Magnusson KR, Hauck L, Jeffrey BM, Elias V, Humphrey A (2015). Relationships between diet-related changes in the gut microbiome and cognitive flexibility. Neuroscience.

[40] Zhu C, Schwarz P, Abakumova I, Aguzzi A (2015). Unaltered prion pathogenesis in a mouse model of high-fat diet-induced insulin resistance. PLoS One.

[41] Prinz M, Huber G, Macpherson AJS, Heppner FL, Glatzel M (2003). Oral prion infection requires normal numbers of Peyer's patches but not of enteric lymphocytes. Am J Pathol.

[42] Horiuchi M, Furuoka H, Kitamura N, Shinagaw M (2006). Alymphoplasia mice are resistant to prion infection via oral route. Jpn J Vet Res.

[43] Glaysher BR, Mabbott NA (2007). Role of the GALT in scrapie agent neuroinvasion from the intestine. J Immunol.

[44] Donaldson DS, Else KJ, Mabbott NA (2015). The gut-associated lymphoid tissues in the small intestine, not the large intestine, play a major role in oral prion disease pathogenesis. J Virol.

[45] Bradford BM, Reizis B, Mabbott NA (2017). Oral prion disease pathogenesis is impeded in the specific absence of CXCR5-expressing dendritic cells. J Virol.

[46] Donaldson DS, Bradford BM, Artis D, Mabbott NA (2015). Reciprocal regulation of lymphoid tissue development in the large intestine by IL-25 and IL-23. Mucosal Immunol.

[47] Kashyap PC, Marcobal A, Ursell LK, Larauche M, Duboc H (2013). Complex interactions among diet, gastrointestinal transit, and gut microbiota in humanized mice. Gastroenterology.

[48] Reikvam DH, Erofeev A, Sandvik A, Grcic V, Jahnsen FL (2011). Depletion of murine intestinal microbiota: effects on gut mucosa and epithelial gene expression. PLoS One.

[49] Lunnon K, Teeling JL, Tutt AL, Cragg MS, Glennie MJ (2011). Systemic inflammation modulates Fc receptor expression on microglia during chronic neurodegeneration. J Immunol.

[50] Cunningham C, Campion S, Lunnon K, Murray CL, Woods JFC (2009). Systemic inflammation induces acute behavioral and cognitive changes and accelerates neurodegenerative disease. Biol Psychiatry.

[51] Sigurdson CJ, Heikenwalder M, Manco G, Barthel M, Schwarz P (2009). Bacterial colitis increases susceptibility to oral prion disease. J Infect Dis.

[52] Donaldson DS, Sehgal A, Rios D, Williams IR, Mabbott NA (2016). Increased abundance of M cells in the gut epithelium dramatically enhances oral prion disease susceptibility. PLoS Pathog.

[53] Valleron A-J, Boelle P-Y, Will R, Cesbron J-Y (2001). Estimation of epidemic size and incubation time based on age characteristics of vCJD in the United Kingdom. Science.

[54] Wilesmith JW (1993). BSE: Epidemiological approaches, trials and tribulations. Prev Vet Med.

[55] Diack AB, Head MW, Mccutcheon S, Boyle A, Knight R (2014). Variant CJD. 18 years of research and surveillance. Prion.

[56] Gill ON, Spencer Y, Richard-Loendt A, Kelly C, Dabaghian R (2013). Prevalent abnormal prion protein in human appendixes after bovine spongiform encephalopathy epizootic: large scale survey. BMJ.

[57] Lorenz RG, Chaplin DD, Mcdonald KG, Mcdonough JS, Newberry RD (2003). Isolated lymphoid follicle formation is inducible and dependent upon lymphotoxin-sufficient B lymphocytes, lymphotoxin β receptor, and TNF receptor I function. J Immunol.

[58] Pabst O, Herbrand H, Friedrichsen M, Velaga S, Dorsch M (2006). Adaptation of solitary intestinal lymphoid tissue in response to microbiota and chemokine receptor CCR7 signaling. J Immunol.

[59] Fraser H, Dickinson AG (1968). The sequential development of the brain lesions of scrapie in three strains of mice. J Comp Pathol.

[60] Mccutcheon S, Langeveld JPM, Tan BC, Gill AC, de Wolf C (2014). Prion protein-specific antibodies that detect multiple TSE agents with high sensitivity. PLoS One.

[61] Inman CF, Rees LEN, Barker E, Haverson K, Stokes CR (2005). Validation of computer-assisted, pixel-based analysis of multiple-colour immunofluorescence histology. J Immunol Methods.

